# Prediction of dengue fever outbreaks using climate variability and Markov chain Monte Carlo techniques in a stochastic susceptible-infected-removed model

**DOI:** 10.1038/s41598-022-09489-y

**Published:** 2022-03-31

**Authors:** Tarun Kumar Martheswaran, Hamida Hamdi, Amal Al-Barty, Abeer Abu Zaid, Biswadeep Das

**Affiliations:** 1 Department of Mathematics, Waterford School, Salt Lake City, UT USA; 2 Department of Biology, College of Science, Taif University, P.O. Box 11099, Taif, 21944 Saudi Arabia; 3 Department of Biology, Alkhormah University College, Taif University, Taif, 21974 Saudi Arabia; 4 School of Biotechnology, Kalinga Institute of Industrial Technology, KIIT Deemed to be University, Bhubaneswar, Odisha India

**Keywords:** Computational biology and bioinformatics, Genetics

## Abstract

The recent increase in the global incidence of dengue fever resulted in over 2.7 million cases in Latin America and many cases in Southeast Asia and has warranted the development and application of early warning systems (EWS) for futuristic outbreak prediction. EWS pertaining to dengue outbreaks is imperative; given the fact that dengue is linked to environmental factors owing to its dominance in the tropics. Prediction is an integral part of EWS, which is dependent on several factors, in particular, climate, geography, and environmental factors. In this study, we explore the role of increased susceptibility to a DENV serotype and climate variability in developing novel predictive models by analyzing RT-PCR and DENV-IgM confirmed cases in Singapore and Honduras, which reported high dengue incidence in 2019 and 2020, respectively. A random-sampling-based susceptible-infected-removed (SIR) model was used to obtain estimates of the susceptible fraction for modeling the dengue epidemic, in addition to the Bayesian Markov Chain Monte Carlo (MCMC) technique that was used to fit the model to Singapore and Honduras case report data from 2012 to 2020. Regression techniques were used to implement climate variability in two methods: a climate-based model, based on individual climate variables, and a seasonal model, based on trigonometrically varying transmission rates. The seasonal model accounted for 98.5% and 92.8% of the variance in case count in the 2020 Singapore and 2019 Honduras outbreaks, respectively. The climate model accounted for 75.3% and 68.3% of the variance in Singapore and Honduras outbreaks respectively, besides accounting for 75.4% of the variance in the major 2013 Singapore outbreak, 71.5% of the variance in the 2019 Singapore outbreak, and over 70% of the variance in 2015 and 2016 Honduras outbreaks. The seasonal model accounted for 14.2% and 83.1% of the variance in the 2013 and 2019 Singapore outbreaks, respectively, in addition to 91% and 59.5% of the variance in the 2015 and 2016 Honduras outbreaks, respectively. Autocorrelation lag tests showed that the climate model exhibited better prediction dynamics for Singapore outbreaks during the dry season from May to August and in the rainy season from June to October in Honduras. After incorporation of susceptible fractions, the seasonal model exhibited higher accuracy in predicting outbreaks of higher case magnitude, including those of the 2019–2020 dengue epidemic, in comparison to the climate model, which was more accurate in outbreaks of smaller magnitude. Such modeling studies could be further performed in various outbreaks, such as the ongoing COVID-19 pandemic to understand the outbreak dynamics and predict the occurrence of future outbreaks.

## Introduction

Dengue fever is a highly debilitating and fatal mosquito-borne arboviral disease (DENV), responsible for over 25,000 deaths annually. It is highly prevalent in the tropical and subtropical areas across the globe; and has recently invaded to new areas, such as the continental United States, where it accounts for over 2.35 million cases each year^1–3^. The World Health Organization (WHO) estimated that more than 40% of the world’s population are at risk of contracting dengue fever, and around 50–100 million new infections occur annually in over 100 countries^4^. During the twenty-first century, climate change and rapidly increasing international travel have considerably increased dengue incidence in the USA and other temperate countries. Furthermore, DENV exhibited a transmission cycle causing repeated outbreak burden to the equatorial and tropical countries from 2000 to 2018, which could be modeled with a trigonometric curve^5,6^. Interestingly, in 2019, there was an unexpected surge in the reported case counts in several countries of Southeast Asia and Latin America, leading dengue to be listed in the top 10 public health threats in the year^7^. In addition, several countries experienced unprecedented 2019–2020 dengue epidemic, which have been reported to have occurred because of changes in the dominant serotype, though the epidemiology was found to be solely climate-related in several Central American countries^8–10^.

Developing a reliable vaccine is cumbersome owing to the existence of four different dengue viral serotypes; each being able to induce a disease-enhancing antibody response against the other three serotypes^11^. Currently, the underlying mechanism of the antibody-dependent enhancement is not clarified, though data indicates that sudden changes in the dominant serotype cause large increases in the case count. Because dengue infections have no specific treatment, effective preventive measures are essential for disease control and management^9,10^. In this regard, early warning system (EWS) pertaining to dengue outbreaks is imperative, and prediction is the integral part of EWS, which is dependent on several factors, in particular climate, geography, and environment. The need for early detection strategies has led to the implementation of several methods for tracking disease transmission. Mathematical models provide a way to understand the dynamics of epidemics as well to help local health organizations and governments implement appropriate public health strategies and ensure optimal use of resources during an epidemic^12,13^. In the past, epidemiologists have used a Susceptible-Infected-Removed (SIR) model^14,15^, incorporating both human and mosquito populations into compartments in ordinary differential equations (ODEs) for simulating the spread of a single strain of the virus. For dengue fever, the susceptible compartment includes those who can possibly fall ill with the dominant strain of the virus for a certain time frame, infected includes those who have contracted the disease, and removed is the compartment for those who have recovered from the strain. Deterministic models are determined using the parameter values and initial conditions. Stochastic models consider the noise, which is characteristic of biological processes^16^. Therefore, stochastic SIR model to dengue fever incidence data could be fitted properly in a Bayesian framework.

Singapore is one of the most dengue-prone areas in the world, located in the Southeast Asian region. Since 1990s, there have been frequent dengue outbreaks in the country. The Singapore Ministry of Health (MOH) took measures after the 2004–2005 epidemic, with the aid of hospitals and primary care centers for improving the management of dengue (Ministry of Health–Singapore, 2007). Few models have been developed to aid the country^17,18^, and even fewer are based on stochastic methods^8^. In 2012, the National Environmental Agency (NEA) began the *Wolbachia* project to release genetically altered male *Aedes* mosquitoes into the environment to mate with female mosquitoes. The eggs of their offspring never hatch because of biological cytoplasmic incompatibility^19^. The method has been said to work up to a certain degree for decreasing case magnitude; however, it has ultimately been ineffective owing to populations returning to pre-introduction levels^20^.

The 2019–2020 dengue epidemic struck Honduras with over 110,000 cumulative cases over the course of 2019, including over 19,000 cases of severe dengue (PAHO/WHO Data- Honduras, 2020). This was the worst outbreak the country had ever experienced, and cautioned much more awareness from the Pan-American Health Organization (PAHO, 2020). Given the rapid increase in case count, the organization speculated that the driving force might include climate change and an increase in the general number of susceptible humans after immune response. Studies have reported the co-circulation of all four dengue virus serotypes in the country, which lays emphasis to provide an early predictive capability for this disease to prevent future outbreaks^22^. Climate influences *Aedes* mosquito development and can modulate virus replication rates. High temperature shorten the extrinsic incubation period and increases the number of mosquitoes, becoming infectious in their lifetime. Rainfall and other moisture indicators provide enhanced breeding habitats, though it has been reported that heavy rainfall can flush out breeding sites of the vectors. Humidity, reported as a percentage, increases the survival rate of mosquitoes as they develop to their biting stage and provides increased appropriate breeding sites for the insects^23^. The El Niño Southern Oscillation (ENSO) is an observed phenomenon in the Pacific Ocean, which begins with a rise in the sea surface temperatures across the Eastern Pacific, extending into the Western Pacific over time. Ultimately, this results in a variety of changes in climate, including higher air temperature, and increased rainfall throughout the world^24^. The relation between the progression of El Nino and La Nina events and dengue incidence has also been explored^25–27^; however, the exact relation with the magnitude of an outbreak is unclear.

In this study, a stochastic SIR model was developed for assessing the transmission of dengue fever, which was additionally taken into account through assessing the data on maximum temperature, minimum temperature, average temperature, precipitation, relative humidity, wind speed, dew point, visibility and sea level pressure. R package *rjags* was utilized to fit the model to dengue case count data and serotype stratification through 2012–2020 retrieved from the public records of Singapore and Honduras. Since the SIR model tracks a single serotype at a time, and dengue outbreaks are typically characterized by distinct serotypes from one year to the next, we fit the model to each outbreak year separately, letting the initial susceptible fraction vary across years for each serotype. As a null model, we assume that transmission is not seasonal, and is constant throughout the season. After estimating susceptible fractions at the start of annual outbreaks, we explore two model extensions. The *seasonal* model assumes that the basic reproduction number of the disease varies sinusoidally throughout the year, while the *climate* model includes statistical relationships between weather and dengue fever with lag times. We fit these models to the data for the 2019/2020 epidemic years and run forward simulations to see how well they reproduce the outbreaks in other years. We further explored whether the strength of ENSO oscillations can predict dengue incidence in Singapore and Honduras.

## Results

### Null model

**Figure 1:**
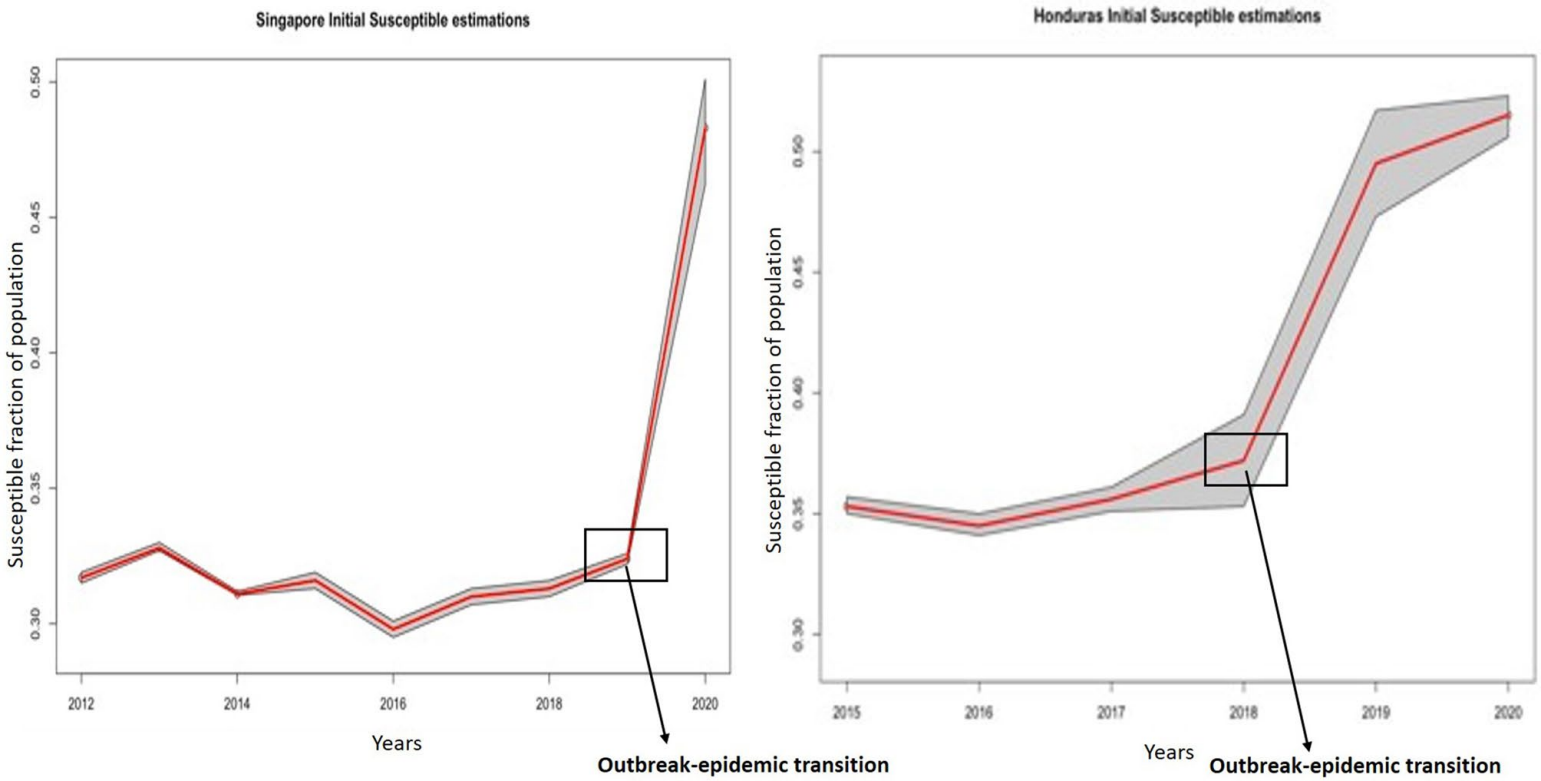
Trend of susceptible fractions by year for Singapore and Honduras. Red line represents point estimates and gray region is 95% confidence interval. The outbreak to epidemic transition is denoted as the position from where steep increase in cases occur, which was 2019–2020 for Singapore and 2018–2019 for Honduras.

Assessment of Singapore 2020 and Honduras 2019 outbreak data with controlling the transmission rate produced a set of parameters and initial population susceptibility fractions, represented as a fraction of the country’s population in the week immediately before the first epidemiological week of a new year (Table 1). The estimated susceptible fractions for each country, by year, are presented in Table 2 and Fig. 1. All else held constant, 48.3% of the Singapore population was estimated as susceptible in 2020, in comparison to 32.4% in 2019, 31.3% in 2018, 31% in 2017, 29.8% in 2016, 31.6% in 2015, 32.3% in 2014, 34% in 2013 and 31.7% in 2012. In Honduras, 51.5% of the population was estimated as susceptible in 2020, with 49.5% in 2019, 37.2% in 2018, 35.6% in 2017, 34.5% in 2016 and 35.3% in 2015.

**Table 1:** Estimates of parameters from large-outbreak fit years for the null model.

Parameter	Singapore 2020 susceptible fraction point estimate (95% CI)	Honduras 2019 susceptible fraction point estimate (95% CI)
S [0]	0.483 (0.462, 0.501)	0.495 (0.473, 0.517)
τ_C_	0.04 (0.02, 0.07)	0.06 (0.01, 0.1)

**Table 2:** Initial susceptible fractions by year for Singapore and Honduras along with 95% CI.

Year	Singapore	Honduras
2012	0.317 (0.315, 0.319)	N/A
2013	0.340 (0.338, 0.342)	N/A
2014	0.323 (0.322, 0.324)	N/A
2015	0.316 (0.313, 0.319)	0.353 (0.350, 0.357)
2016	0.298 (0.295, 0.301)	0.345 (0.341, 0.35)
2017	0.310 (0.307, 0.313)	0.356 (0.351, 0.361)
2018	0.313 (0.310, 0.316)	0.372 (0.353, 0.391)
2019	0.324 (0.322, 0.326)	FIT
2020	FIT	0.515 (0.506, 0.523)

*N/A* not applicable, *Fit* years of fit for the mod.

### Seasonal model

**Figure 2:**
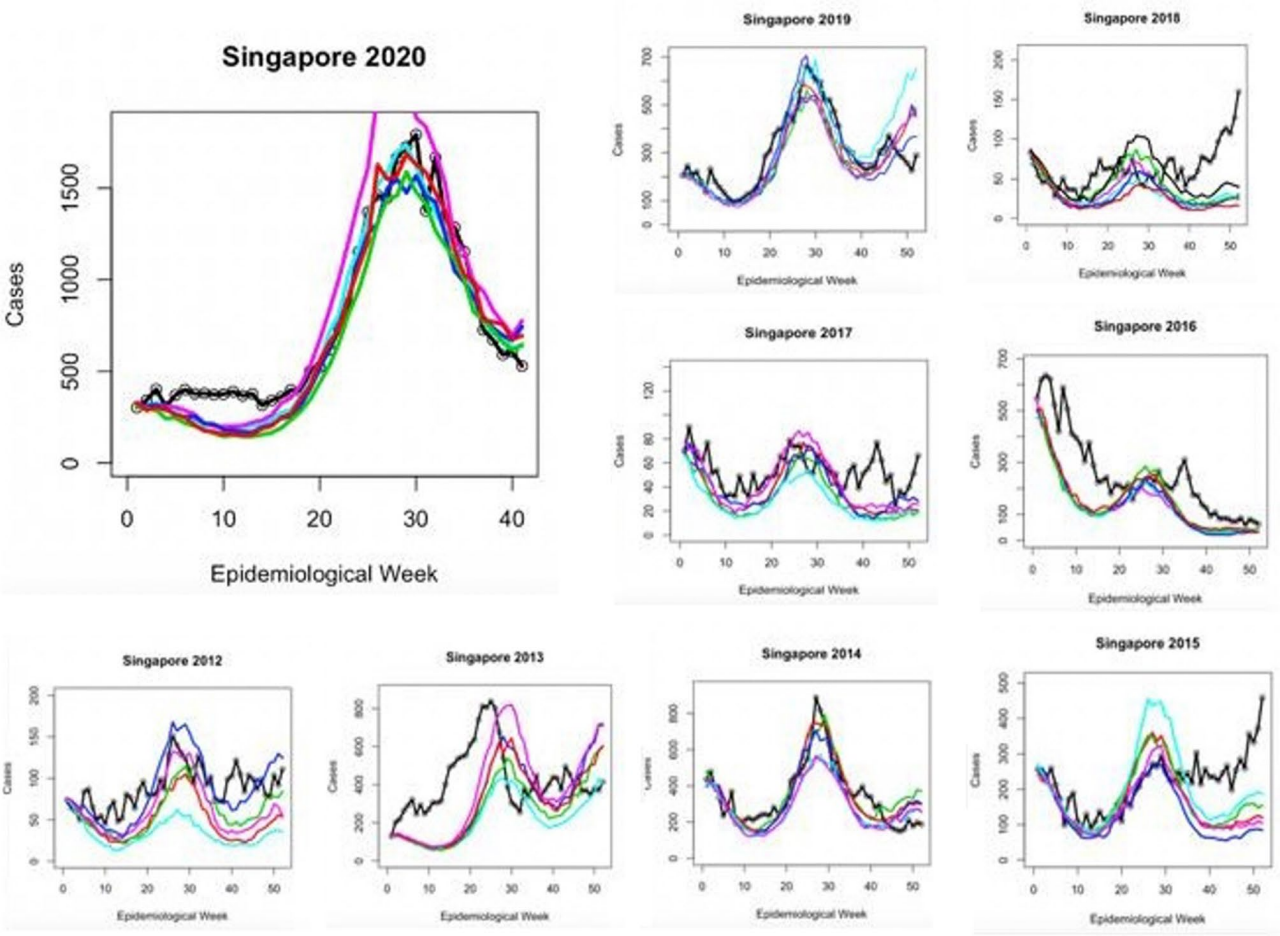
Graph exhibiting the results of seasonal model testing for Singapore yearly outbreaks. Colored lines represent five different forward simulation runs. Solid black line is the reported case count for each year.

**Figure 3:**
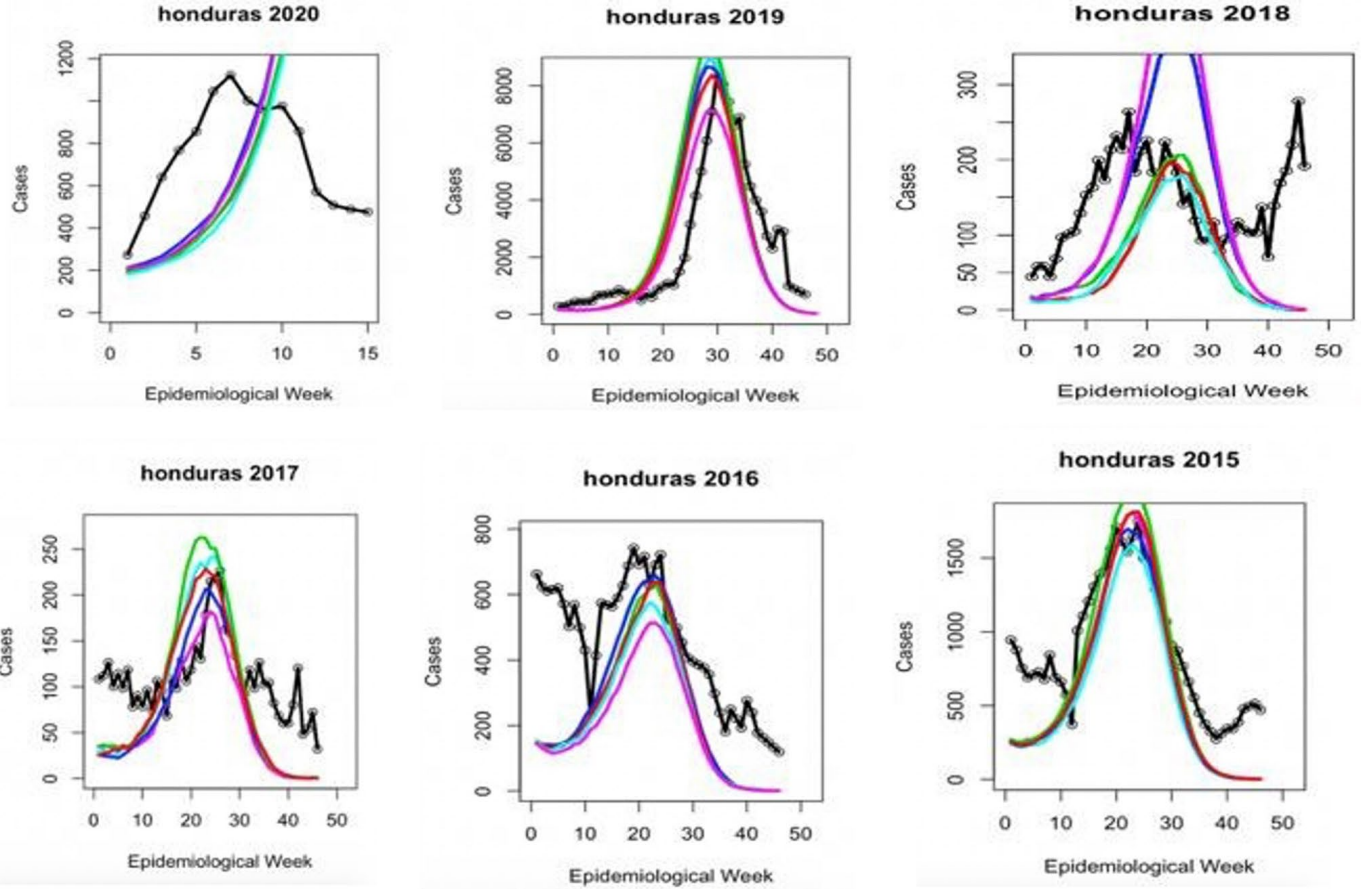
Graph exhibiting the results of seasonal model testing for Honduras yearly outbreaks. Colored lines represent five different forward simulation runs. Solid black line is the reported case count for each year.

The results from the seasonal model, incorporated with transmission rate estimates are presented in Table 3. Cross-correlation demonstrated that there was a 5 week lag effect in Singapore between an increase in transmission rate and a case increase, and a 6 week lag effect in Honduras. The results of five forward simulations per outbreak year using the produced seasonal beta regression are displayed in Figs. 2 and 3, and were statistically verified using Pearson’s correlation values (Table 4). On average, the seasonal model explained 98.5% of variance in Singapore 2020 cases, 83.1% in 2019, 7% in 2018, 41.8% in 2017, 44.3% in 2016, 42.4% in 2015, 74.2% in 2014, 14.2% in 2013 and 57.7% in 2012. Lag between forward simulations and the case report was shown to vary from − 6 to + 4 weeks. For Honduras case reports, the seasonal model explained 18.6% variance in 2020, 92.8% in 2019, 21.3% in 2018, 57.5% in 2017, 59% in 2016, 91% in 2015.

**Table 3:** Parameter estimates for the trigonometric seasonal models for beta shown for both countries.

Country	Parameter	Point estimate: mean (95% CI)	Country	Parameter	Point estimate: mean (95% CI)
Singapore	U	2.00 (1.99,2.01)	Honduras	U	2.03 (1.37,2.66)
Singapore	A_1_	0.41 (0.37,0.46)	Honduras	A_1_	2.01 (1.93, 2.20)
Singapore	A_2_	− 0.39 (− 0.41, − 0.38)	Honduras	A_2_	− 2.92 (− 3.32, − 2.15)
Singapore	T_1_	26.03 (25.02,27.56)	Honduras	T_1_	51.73 (50.19,52.48)
Singapore	T_2_	51.82 (50.19,54.48)	Honduras	T_2_	51.68 (50.22,53.63)
Singapore	Lag	5 weeks	Honduras	Lag	6 weeks

**Table 4:** Results of statistical testing for seasonal models.

Country	Year	Average R^2^ value	Autocorrelation lag	Country	Year	Average R^2^ value	Autocorrelation lag
Singapore	2012	0.577	− 2	Honduras	2012	N/A	N/A
Singapore	2013	0.142	− 6	Honduras	2013	N/A	N/A
Singapore	2014	0.742	0	Honduras	2014	N/A	N/A
Singapore	2015	0.424	+ 4	Honduras	2015	0.910	0
Singapore	2016	0.443	+ 2	Honduras	2016	0.595	− 4
Singapore	2017	0.418	− 1	Honduras	2017	0.575	+ 2
Singapore	2018	0.070	− 2	Honduras	2018	0.213	− 10
Singapore	2019	0.831	0	Honduras	2019	0.928 (fit)	0
Singapore	2020 (fit)	0.985	0	Honduras	2020	0.186	− 5

"Average" refers to the average of five displayed forward simulations. Lag times were rounded to nearest whole number if result was a decimal.

### Climate model

**Figure 4:**
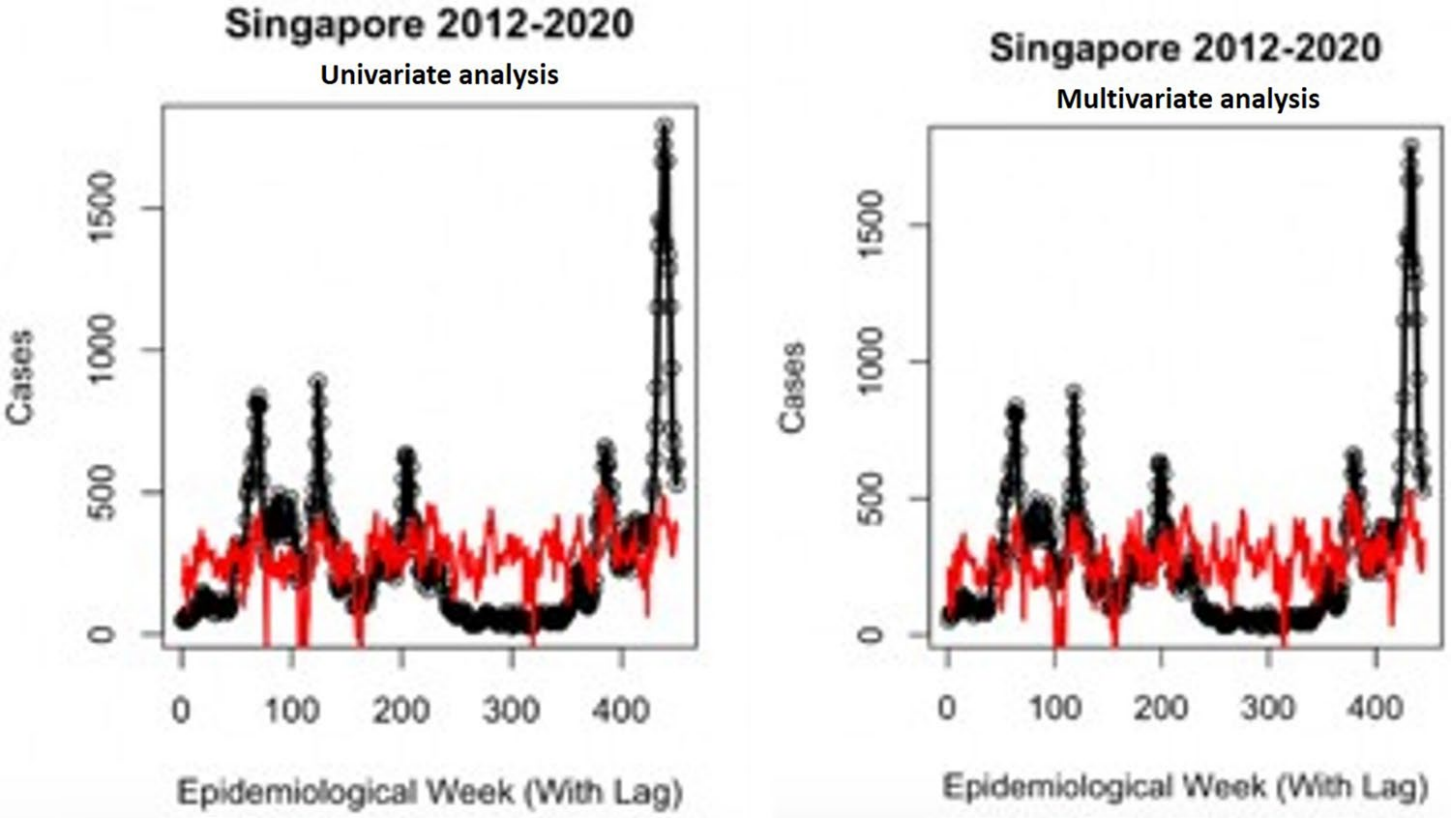
Graph showing Singapore univariate and multivariate climate regressions. Singapore univariate model utilizes dew point, while multivariate model additionally adjusts for wind speed and maximum temperature. Colored line indicates prediction and the scatter plot indicates the reported cases.

**Figure 5:**
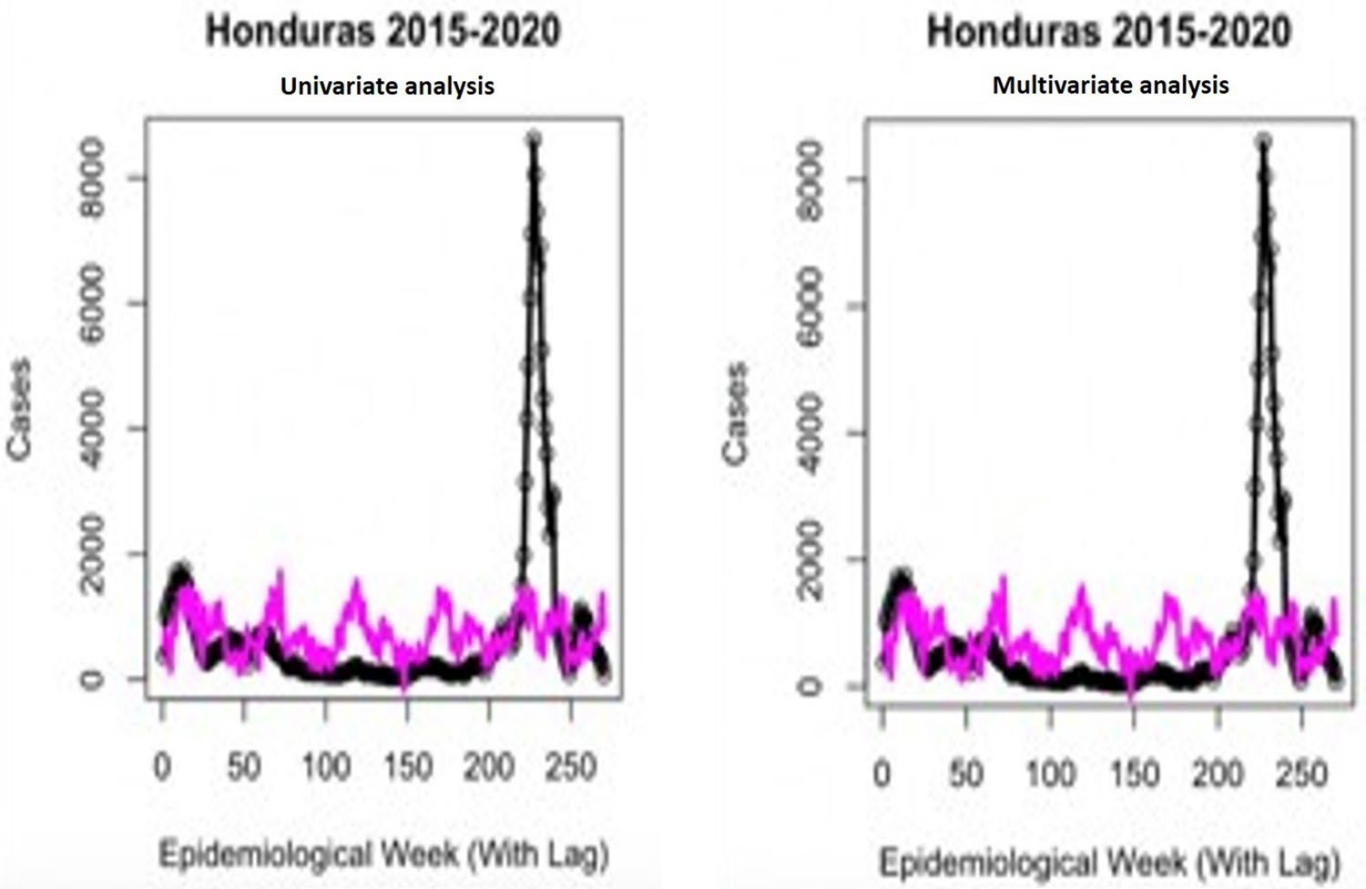
Graph showing Honduras univariate and multivariate climate regressions. Honduras univariate model utilizes relative humidity, while multivariate model additionally adjusts for minimum temperature, maximum temperature and dew point. Colored line indicates prediction and the scatter plot indicates the reported cases.

**Figure 6:**
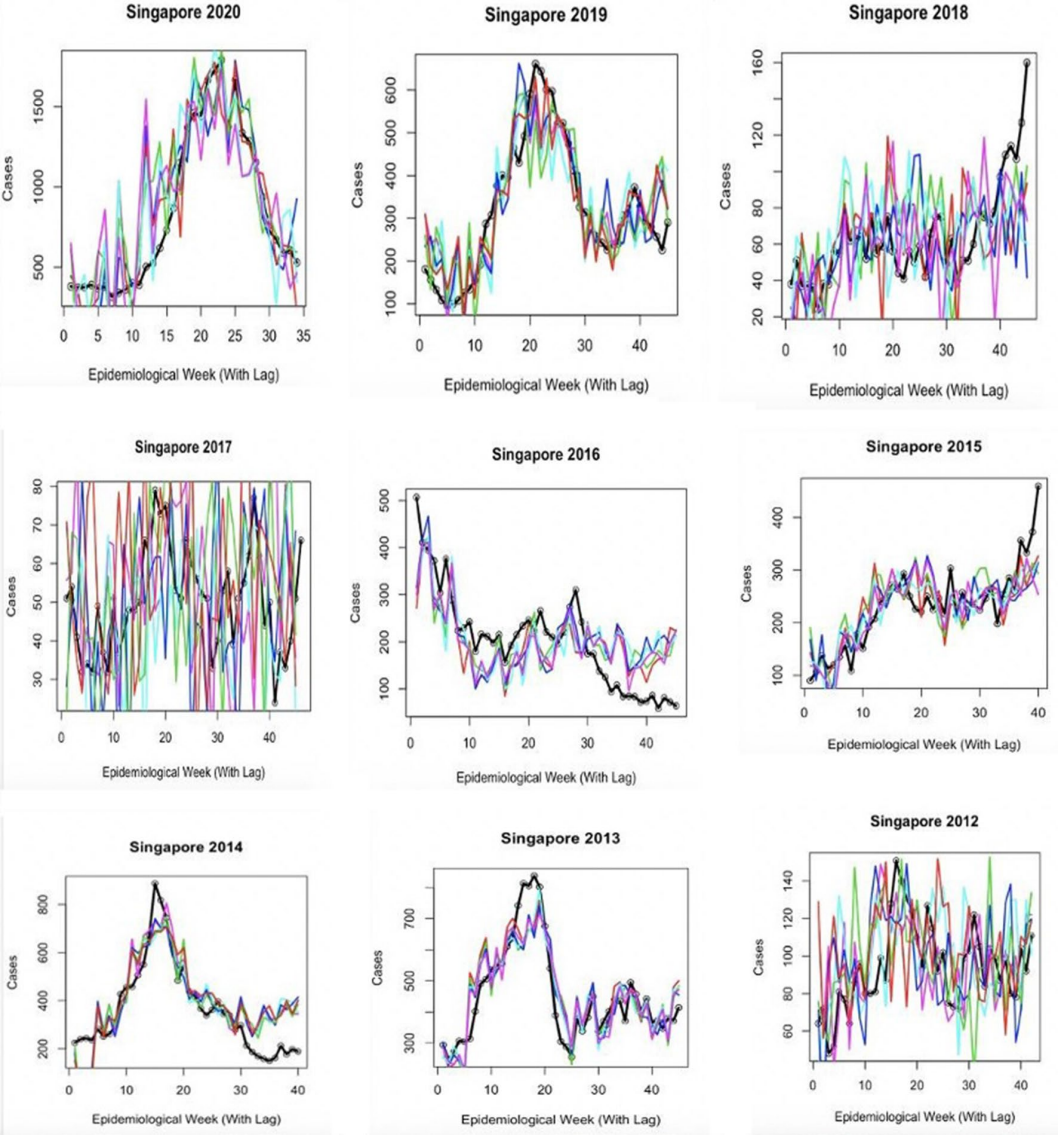
Graph showing the results of climate model testing (with lag) for Singapore model from 2012 to 2020. Colored lines represent five different forward simulation runs. Solid black line is the reported case count for each year.

**Figure 7:**
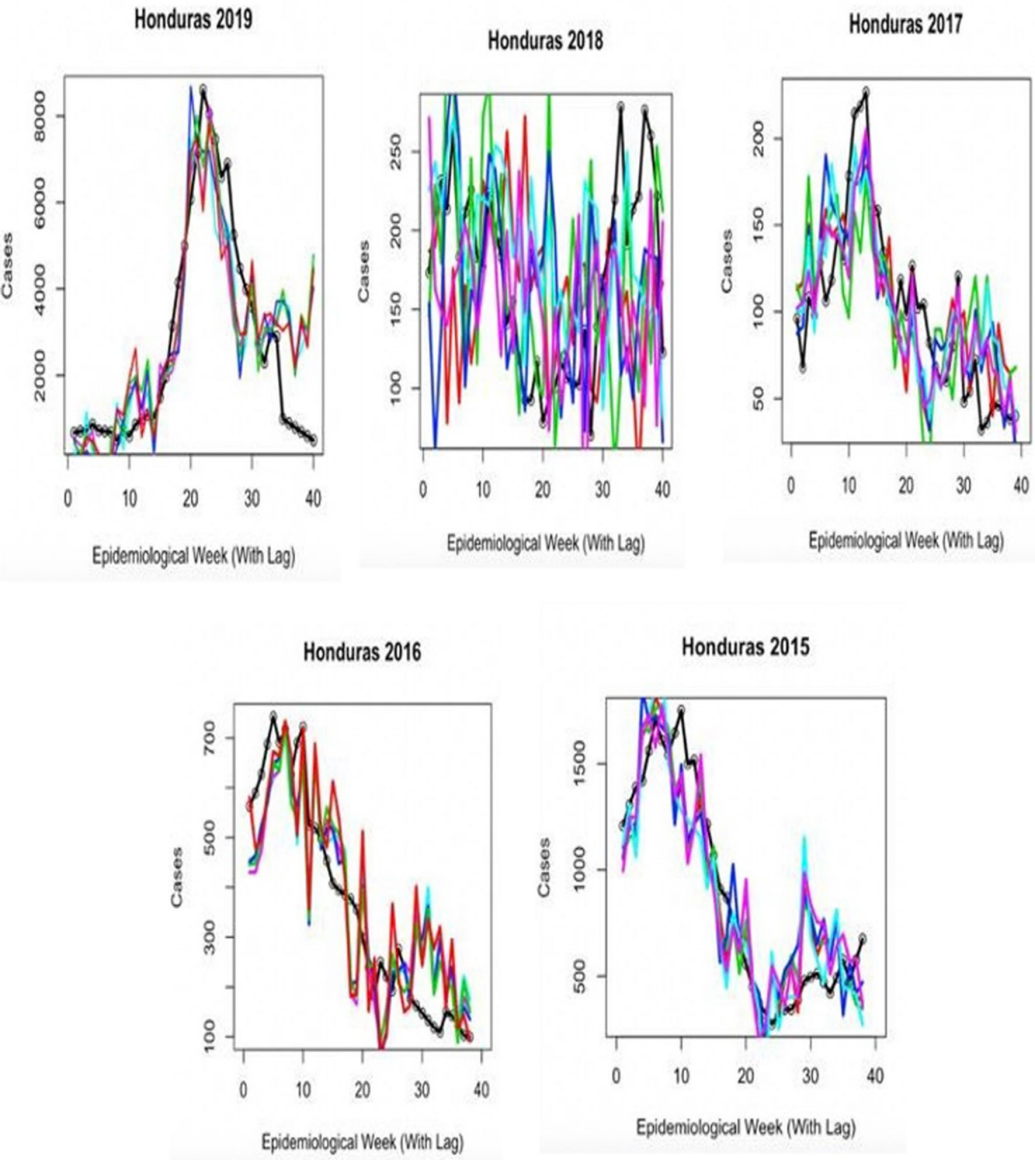
Graph showing the results of climate model testing (with lag) of climate model testing for Honduras model from 2015 to 2019. Colored lines represent five different forward simulation runs. Solid black line is the reported case count for each year.

The correlation results between El Nino measures and weekly incidence data are summarized in Table 5, with p values from each regression displayed. For the Southern Oscillation Index (SOI) measure in Singapore, all regression types were significant, however the strongest significance was with an exponential relationship (lag = 42 weeks, p = 5.51e−10). For the Oceanic Nino Index (ONI) measure in Singapore, all regression types were again significant, but the general additive model displayed the strongest results (lag = 20 weeks, p = 6.28e−22). Honduras El Nino measures reflected similar relationships, with the exponential model for SOI performing strongest (lag = 19 weeks, p = 4.56e−17) and the general additive model for ONI (lag = 42 weeks, p = 2.08e−22). The results for correlating weekly climate data and case count are summarized in Table 6 for tests with linear, quadratic exponential and general additive models. The most significant value is displayed for each variable from testing with 1–12 weeks of lag as well as the type of regression that was optimal. The top performing univariate and multivariate model fits for Singapore and Honduras are presented in Figs. 4 and 5, relating to their weekly reported cases. Finally, the results from forward simulations with the climate model are presented in Table 7 and Figs. 6 and 7 for both the countries. On average, the model explained 75.3% of variance in Singapore 2020 cases, 71.5% in 2019, 43.3% in 2018, 30.1% in 2017, 42.2% in 2016, 62.2% in 2015, 65.5% in 2014, 75.4% in 2013 and 32.9% in 2012. Lag between forward simulations and the case report was shown to vary from − 10 to + 1 weeks for both the countries. It should be noted that − 10 is a clear outlier, and all other lag values lie between − 2 and + 1 week. For Honduras case reports, the climate model explained 68.3% in 2019, 16.2% in 2018, 68.3% in 2017, 72.4% in 2016, 74.3% in 2015 (Table 7).

**Table 5:** El Niño Index correlation with reported case data in both countries.

Singapore	Southern oscillation index (SOI)	Oceanic nino index (ONI)
Linear, best lag	44	35
p-value	1.03e−04	1.67e−05
Quadratic, best lag	16	40
p-value	7.98e−06	4.52e−09
Exponential, best lag	42	31
p-value	**5.51e**−10***	5.62e−13
General additive, best lag	16	20
p-value	5.25e−07	**6.28e**−22***
**Honduras**		
Linear, best lag	12	32
p-value	3.58e−05	9.13e−03
Quadratic, best lag	10	44
p-value	9.18e−06	2.03e−07
Exponential, best lag	19	18
p-value	**4.56e**−17***	1.39e−14
General additive, best lag	4	42
p-value	1.71e−10	**2.08e**−22***

Significance values are in bold.

**Table 6:** Weekly climate correlation results with reported case data in both countries.

Singapore	Max Temp	Min Temp	Visibility	Cum Rain	Min Temp	Wind	Sea level	Ave temp	Dew	Precipitation	Relative humidity
Linear, best lag	12	5	11	5	11	9	4	12	6	5	5
p-value	1.06e−09	**1.68e**−02**	**1.41e**−**01**	**1.09 e**−**01**	1.86e−02	6.78e−05	**3.75e**−03***	2.27e−06	6.47e−14	**1.09e**−**01**	1.68e−02*
Quadratic, best lag	12	5	12	5	6	9	3	12	7	5	5
p-value	**2.3e**−14***	5.16e−02	2.41e−01	2.13 e−01	3.14e−02	2.25e−04	9.04e−03	**6.88e**−07***	**5.82e**−21***	2.13e−01	5.16e−02
Exponential, best lag	12	12	12	5	2	9	12	11	6	5	12
p-value	6.29e−06	1.95e−02	1.45e−02	4.67e−01	**2.58e**−03**	3.8e−02	2.14e−02	5.67e−05	2e−09	4.67e−01	1.95e−02
General additive, best lag	12	5	11	5	9	9	4	12	7	5	5
p-value	8.32e−13	**1.68e**−02**	**1.41e**−**01**	**1.09 e**−**01**	4.75e−02	**6.75e**−05***	6.21e−03	6.05e−06	7.75e−21	**1.09e**−**01**	1.68e−02*
**Honduras**											
Linear,best lag	1	12	12	12	6	5	10	8	12	12	12
p-value	1.36e−03	1.39e−05	5.23e−04	3.92e−01	2.33e−01	3.16e−02	3.5e−02	1.7e−02	7.79e−03	3.92e−01	1.39e−05
Quadratic, best lag	1	12	12	12	1	2	10	1	1	12	12
p-value	4.06e−03	4.13e−05	5.97e−04	6.46e−01	6.98e−02	4.1e−02	4.87e−02	5.78e−03	1.41e−02	6.46e−01	4.13e−05
Exponential, best lag	1	11	2	11	12	1	12	1	12	11	11
p-value	**2.36e**−08***	**1.74e**−08***	1.18e−01	**3.56 e**−03***	**1.57e**−02**	**2.52e**−04***	2.25e−01	**2.19e**−05***	**8.36e**−06***	**3.56e**−03**	1.74e−08***
General additive, best lag	10	11	12	12	1	6	9	1	2	12	11
p-value	2.14e−03	4.63e−05	**5.78e**−06***	3.92 e−01	1.06e−01	6.57e−02	**5.13e**−03**	1.54e−02	4.5e−03	3.92e−01	4.63e−05

Bold values represent the highest significance as a predictor for each variable in each country. Lag is in weeks *, < 0.05; **, < 0.01; ***, < 0.001.

*Max* maximum, *Min* minimum, *Cum.* cumulative, *Dew* dew point.

**Table 7:** Statistical results for the climate model.

Country	Year	Average e^E^value	Average autocorrelation lag	Country	Year	Average e^E^value	Average autocorrelation lag
Singapore	2012	0.329	− 10	Honduras	N/A	N/A	N/A
Singapore	2013	0.754	− 2	Honduras	N/A	N/A	N/A
Singapore	2014	0.655	0	Honduras	N/A	N/A	N/A
Singapore	2015	0.622	0	Honduras	2015	0.743	0
Singapore	2016	0.422	+ 1	Honduras	2016	0.724	0
Singapore	2017	0.301	0	Honduras	2017	0.683	0
Singapore	2018	0.433	+ 1	Honduras	2018	0.162	− 2
Singapore	2019	0.715	+ 1	Honduras	2019	0.683 (fit)	0
Singapore	2020	0.753 (fit)	0	Honduras	2020	N/A	N/A

"Average" represents the average correlation value for the five displayed simulations. “Fit” represents the outbreak year the model was fit to.

*N/A* not applicable.

## Discussion

**Figure 8:**
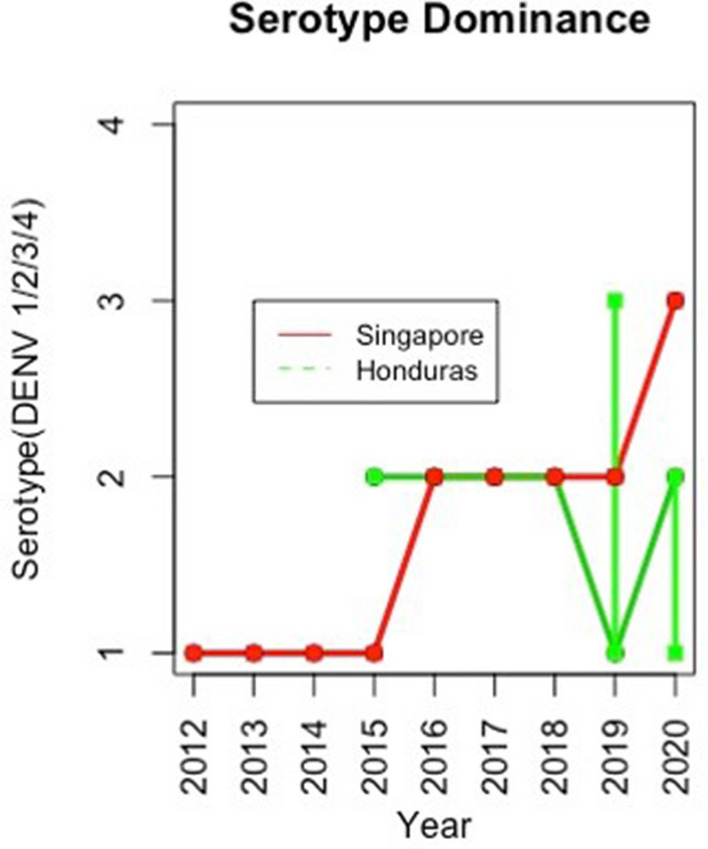
Graph showing the serotype-dominance through 2012–2020 (Singapore) and 2015–2020 (Honduras) representing the circulation of different dengue virus serotypes.

Early disease prediction using mathematical models are currently one of the growing approaches for infectious disease management in the community. Mathematical models are useful for understanding the patterns that infectious diseases follow, and in assisting disease control strategies^34^. Importantly, the model should take into account the complex epidemic dynamics during risk assessment of vector-borne diseases^18^. A model including seasonal factors would be one of the best methods for analyzing infectious diseases such as dengue; however, owing to significant variations in the environmental events and climate change that affect the vectors carrying the pathogens, it is imperative to include additional determinants for the assessment^35^. Therefore, including seasonality in the transmission is essential to reproduce disease incidence accurately. Our results suggest that the size of a given dengue outbreak is primarily determined by the seasonal trend and the type of serotype being circulating in the community (Fig. 8). The graphs showed a strong correlation and matched the magnitude and timing of the peak of the outbreak (Figs. 3, 4, 5, 6, 7). Statistical tests showed that 98.5% of the variance in the case count of Singapore 2020 outbreak could be explained by the trigonometric seasonal model for the transmission parameter in the SIR system. Similarly, 92.8% of the variance in the case count of Honduras 2019 outbreak could be explained by the seasonal model (Table 4). The climate model of transmission reproduced some of the features of the seasonal model; however with some background interference. It would be therefore interesting to investigate the underlying reasons for more noise-dominated seasonal models in 2012, 2017, and 2018 for Singapore, and 2018 for Honduras. Our results also suggested that the size of a given dengue outbreak is primarily determined by the level of immunity to the invading serotype. For example, the seasonal model was ineffective in 2013 Singapore outbreak, wherein only 14.2% variance in case count was explained, in comparison to 75.4% from the climate model (Tables 4 and 7). Interestingly, we found that during the outbreak seasons where the seasonal model failed to reproduce the case counts, the climate model performed better, and vice versa. This suggested that perturbations in the weather can disrupt the environment enough to make predictions from the seasonal model invalid. On the other hand, variation in the weather is not always by itself a reliable indicator for the variability in dengue transmission over the year^36^. The best option would be to extend our model to include an underlying seasonal trend over a year, in addition to accounting for the variation in the weather at a weekly basis.

Our single-strain model leads to some simplifying assumptions. All humans and serotypes are considered heterogeneous and this model did not take into account the immunity for certain strains in a region, or the vaccinated portion of the population, or the rate at which those who are vaccinated lose the protection, all of which can act as a confounding factor. Though all four serotypes are circulating throughout the region at any given time, a dominant serotype is usually dominant for a specific time period. It is important to note that studies have found that the reproduction rates are similar among the four serotypes^37^, which satisfies the assumptions of our model. This phenomenon has occurred in Singapore in recent years with a rise in the circulation of DENV-2 in late 2015, and a shift to DENV-3 in early 2020^10^. Researchers have explored expansion to a multi-serotype model^38^, though the system becomes much more complex to track, in particular when combining the seasonal and climate components. The stochasticity added in the model allows us to observe noise during the assessments and revealed the differences in simulation potential under a given set of initial conditions. The null model transmission parameter estimates were based on reliable measures gathered from several sources and studies; however they may not represent the exact basic reproduction number of the disease. Hence, we implemented sampling from a random distribution at each timestep in hopes of capturing a reliable estimate of the initial susceptible fraction.

The seasonal model worked statistically and visually well to show the peak of an outbreak occurring during a specific season. With the input of the final conditions of reported cases and estimated susceptible population of previous year, we can successfully predict the progress of the outbreak in the year. The lag between an increase in the transmission and an increase in the incidence is significant for many reasons. Intervention strategies put into place by government organizations can have a time frame for assessing the effectiveness of their efforts. Decreasing mosquito breeding rates or enacting chemical spray techniques will lead to a decrease in transmission, leading to a decrease in the case count in near future. The lag also served well in creating models that estimate the association between climate change and the actual transmission parameter of the SIR model. The climate model, such as several other past studies^14,39^, employs the use of lag time between climate variation and increased case count. Along with contrasting assigned relationships between the climate variables, we tested four prominent regression models and obtained significant results. El Niño indicators were assessed; however, they were not significant in multivariable models. These indicators have been shown to have relationships with dengue cases, and the most recent El Niño event ended in August 2019.

Significant variables for Singapore case counts were a combination of average dew point, maximum temperature, and wind speed, with lags and model types shown in Table 6. Honduras climate significance was shown with a combination of relative humidity, minimum temperature and dew point. Dew point is a measure of moisture in the air, which creates optimal environments for mosquitoes; however, this variable has rarely been explored. In addition, humidity parameters were observed to follow annual and sub-annual periodicities^40^, which indicated that this can be safely used as a predictive measure for dengue forecasting. Temperature can have various effects on the mosquito populations; extreme temperatures can decrease breeding sites and populations. Similar to dew point, relative humidity has been shown to significantly increase the oviposition rate among *Aedes* female mosquitoes^41,42^. Once multivariate climate models were put into the SIR model, through lag to the transmission parameter, and the models were adjusted for the initial susceptible fraction, they generated much better results, indicating the precise time of outbreak (Figs. 4 and 5, Table 7). Future work should explore the differences in lag and climate variable significance in other countries that routinely experience outbreaks. Vigilance and caution should be exercised in countries that are at risk of such unexpected epidemics, owing to the expansion and shift of the habitable regions of the vector mosquitoes, along with international solidarity to combat bigger losses. The fact that there are several countries that are already experiencing deaths associated with dengue following the recent outbreaks suggests that more attention should be made on the formation of highly sensitive and specific forecasting techniques along with early detection strategies in clinical practice.

**Figure 9:**
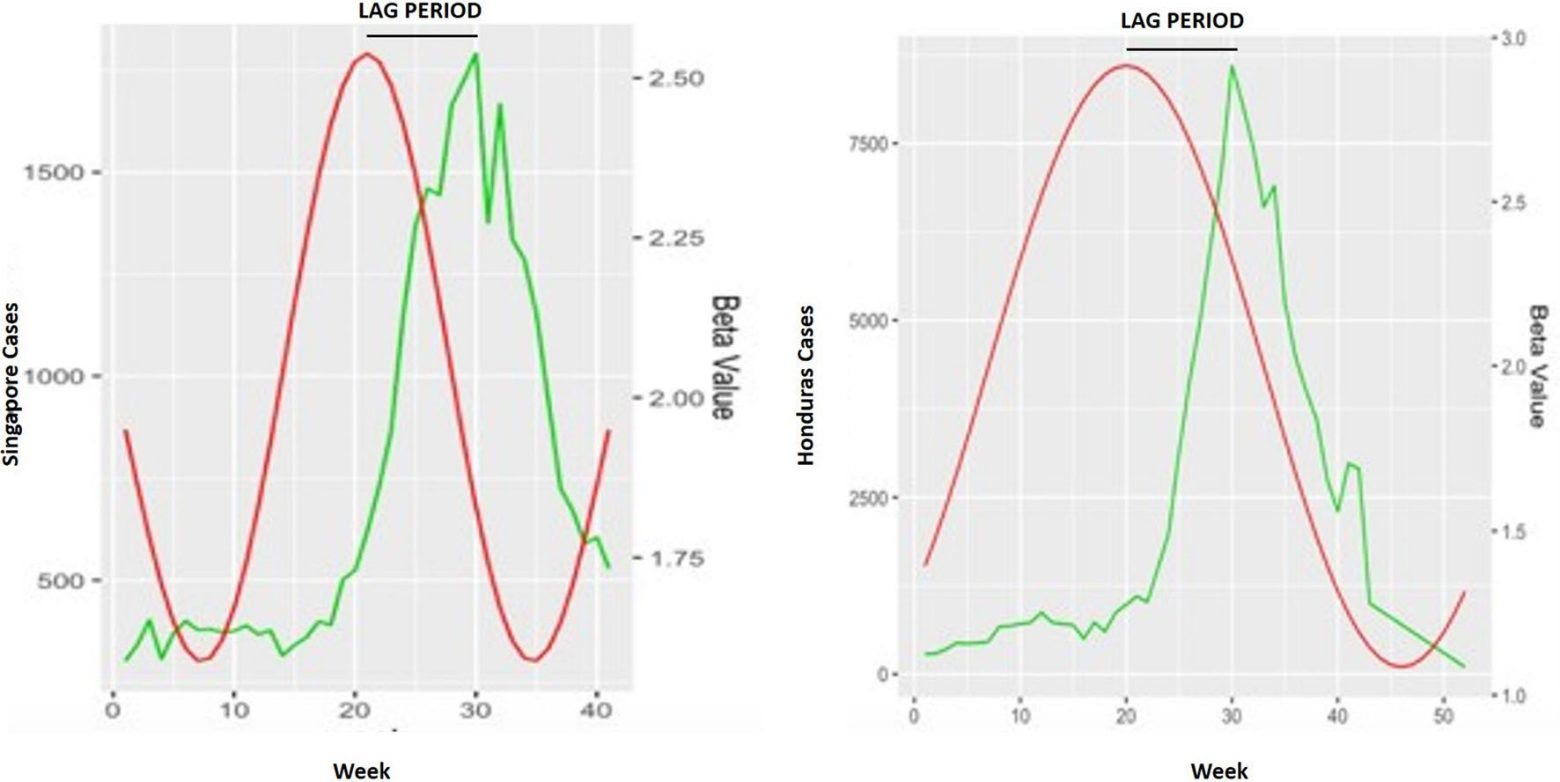
Graph showing the null model analysis exhibiting the trigonometric beta estimates (red) and the cases (green) for Singapore and Honduras. The lag period of about 12–14 weeks is shown for both the countries.

The results in Figs. 2 and 9 tracks approximately what we would expect, with a large jump in the recent 2019/2020 outbreaks. The increase in the susceptible fraction was approximately 49% in Singapore and 33% in Honduras based on our available data (Tables 1 and 2); however, this may vary based on the past prevalence of the serotype. Singapore had not seen an outbreak comprising DENV3 in thirty years (Ministry of Health, 2020), which explains the unprecedented increase in 2020. It should be further noted that including the complete continuous seroprevalence history of the population over the study period could indicate more plausible estimates of susceptibility among the population. This may be because of vaccines being assigned at unknown times, age, and other factors, which could be a limitation of the study. However, given the unconfirmed longevity of dengue vaccines that are still under research pursuit, and owing to the lack of continuous seroprevalence data, generating this estimate through combinatorial approaches of using several models serves as a reliable way to observe how general increased susceptibility plays a role in promoting outbreaks. From our work, these estimates, combined with incorporation of climatic/seasonal influences were able to recreate the 2019–2020 epidemic effectively, while also replicating previous years with high accuracy. In addition, as a retrospective look with our current situation, using 2019–2020 as fit years to determine these susceptible fractions and form other predictive models may lead to some issues. In a general sense, COVID-19 might have caused challenges within the healthcare system including case notification issues that was observed with omission of 2020 Honduras data. Future work should focus on how the presence of COVID and other febrile diseases might have an influence on the models.

## Materials and methods

### Dengue incidence data

**Figure 10:**
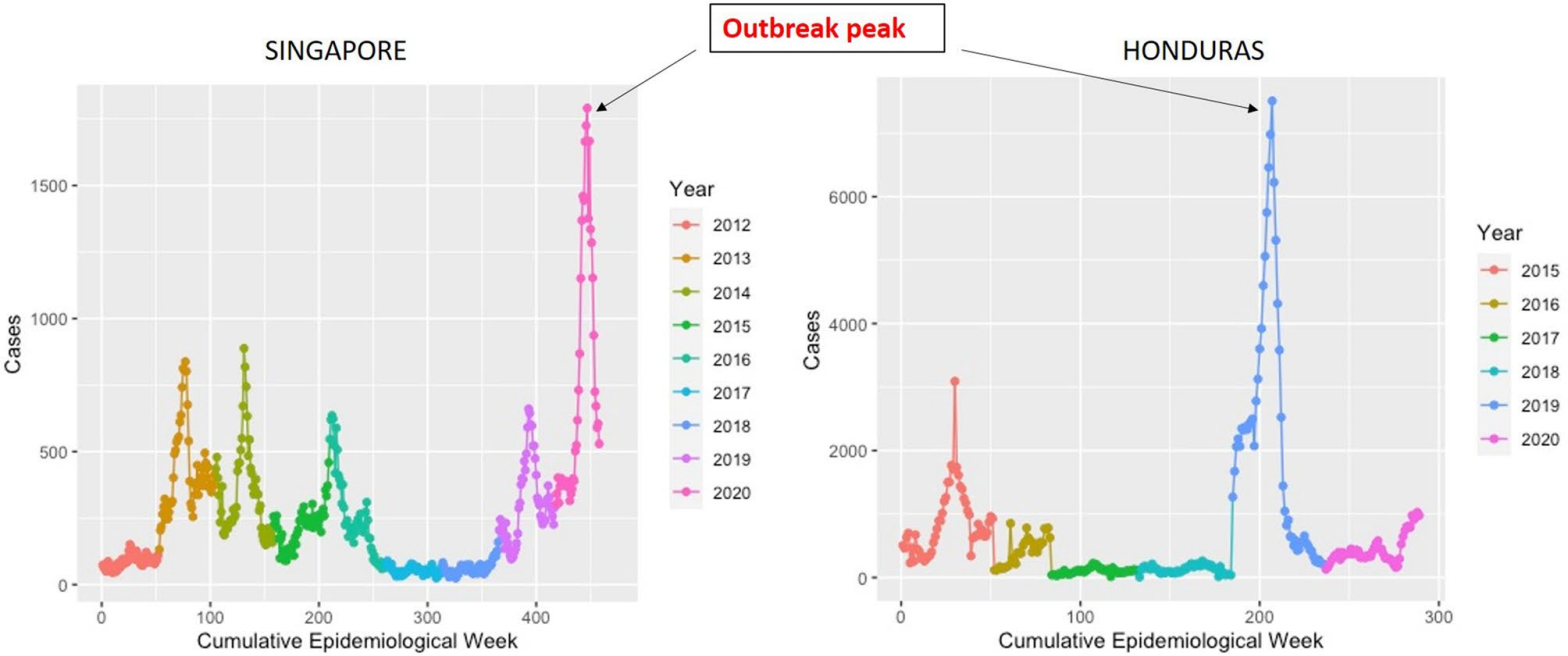
Graph showing the confirmed dengue cases (RT-PCR and DENV-IgM positive) in Singapore and Honduras from 2012 to 2020. Different years are represented with colors as shown in the figure. The peak outbreak period, 2019 for Honduras and 2020 for Singapore is denoted in the pink and blue, respectively.

Dengue incidence data were obtained from the Ministry of Health Infectious Disease Bulletin of Singapore, which publicizes statistics on infectious diseases at the regional level (Ministry of Health 2020). The case count is reported weekly for dengue fever and DHF, further confirmed by RT-PCR and DENV-IgM enzyme linked immunosorbent assay, and visualized by country (Fig. 10). The records currently dates back to 2012 including all 52 weeks of the study period. Weekly case reports of dengue RT-PCR and DENV-IgM confirmed cases in Honduras were also obtained for the period 2015–2020 from the Pan American Health Organization (Pan American Health Organization 2020), which reports weekly dengue cases in Central American countries. For Singapore, annual serotype-specific case data were obtained from National Environment Agency, Singapore and SEARO, WHO. For Honduras, serotype-specific case data were obtained from PAHO, WHO (https://ntdhq.shinyapps.io/dengue5/) and from reports that have cited an increase in the circulation of dengue serotypes at different times^30^. Demographic data for Singapore and Honduras were obtained for the given period. Population data during the period 2013–2019 are available from Singapore’s Department of Statistics (Statistics Singapore—Latest Data—Births & Deaths, 2013) and from the Instituto Nacional de Estadistica (INE), Honduras (Instituto Nacional De Estadistica 2020). Fit years were selected by picking the most recent year for each country that had a sufficient amount of data, which was 2020 for Singapore and 2019 for Honduras. Such selection was because of our specific focus on the 2019–2020 dengue epidemic, and how it relates to predicting past outbreaks. With a specific initial focus on recent years, we can observe how parameter estimations might have changed up to the current day, potentially deducing a cause for the epidemic.

### El Niño and meteorological data

Two indices were observed for the occurrence of El Niño and La Niña: Southern Oscillation Index (SOI), which measures the difference in sea level pressure, and the Oceanic Nino Index (ONI), which measures sea surface temperature. These were manipulated to correlate with weekly reported cases by keeping the monthly value constant across the corresponding epidemiological weeks. These were tested with a lag of 0–46 weeks, which was found to be significant in a previous study^30^. Weekly climate data recordings included measures that were recorded as influencers of the breeding activity of *Aedes* mosquitos, or on an increase in the number of breeding sites. The data were obtained from CustomWeather, a company involved in the World Meteorological Association (WMA) network. The collected measures were maximum temperature, minimum temperature, average temperature, precipitation, average relative humidity, average wind, average dew, average visibility, and average sea level pressure. Each of these was measured daily in Singapore (2012–2020) and Honduras (2015–2020), and a weekly average was taken to match the dengue data and NEA published dates of the epidemiological weeks.

### Stochastic SIR model

A stochastic SIR model (equation set 1) simulated in discrete-time was formulated^32^ and cited references for similar models]. Homogeneous mixing was assumed for all individuals and a time step of 1 week was employed to match the temporal resolution of the incidence data and the average duration of dengue infection. The parameters for the single-serotype SIR model are demonstrated in Table 8. The values of the population varied by location, and the recovery rate and transmission probability were estimated from JAGS based on prior distributions from previous literature.

1 $$ \begin{gathered} \frac{{{\text{dS}}}}{{{\text{dt}}}} = - \frac{{{\upbeta } \cdot {\text{I}} \cdot {\text{S}}}}{{\text{N}}} \hfill \\ \frac{{{\text{dI}}}}{{{\text{dt}}}} = \frac{{{\upbeta } \cdot {\text{I}} \cdot {\text{S}}}}{{\text{N}}} - {\upgamma } \cdot {\text{I}} \hfill \\ \frac{{{\text{dR}}}}{{{\text{dt}}}} = {\upgamma } \cdot {\text{I}} \hfill \\ {\text{C}} = {\rho *} \cdot {\text{I}} \hfill \\ \end{gathered} $$

where N is the total population, S is the susceptible fraction of population, I is the dengue infected and population, C is the confirmed DENV RT-PCR and DENV-IgG case count, γ is the recovery rate, $\beta$ is the transmission parameter and ρ is the reporting rate of DENV infection.

**Table 8:** Parameters of the SIR model that had known values and were not estimated using JAGS.

Parameters	Meaning	Value	Sources
N	Human population	Varies: approximately 4,000,000 in Singapore, 5,000,000 in Honduras	Singapore Department of Statistics (INE)
γ	Recovery rate	1 week*	[34] in references
C	Reported cases	Varies	MoH Infectious Disease Bulletin/PAHO
ρ	Reporting rate	1:6	Tan 2019
N [0]	Population in week before year starts (can be predicted with birth rate)	Varies year to year	Singapore Dept. of Statistics (INE)

*MOH* ministry of health, *Dept.* department, *INE* Instituto Nacional de Estadistica.

### Markov chain Monte Carlo (MCMC) method

MCMC sample parameter values (typically analytically intractable) were utilized from the posterior distribution of the mathematical model, conditional on some specified prior density, allowing us to perform Bayesian inference on the model parameters; thereby, quantities such as the Maximum a Posteriori (MAP) estimates and variance in the parameter could be obtained. Bayesian inference requires the specification of a prior distribution, which is informed by dengue modeling related literature and pre-existing data. We used JAGS^28,29^, a clone of OpenBUGS, with the ability to analyze all examples provided in this classic software. The package “*rjags*” provides compatibility between JAGS and R, and the package “coda” was used for representing parallel runs of our input number of chains for the model (Comprehensive R Archive Network 2019). With JAGS, a monitor called “trace monitor” records the sampled values of the parameters for every nth iteration that is specified^28^. We generated a 95% confidence interval for all SIR parameter values considering mean and variance at each time step. After being fit to country-specific outbreak years, the generated parameter values were then used as input for simulations of future outbreaks in both Singapore and Honduras.

### JAGS model

At each time step, stochasticity is introduced by drawing the state variables susceptible fraction (S), DENV infected population (I), and R from a binomial distribution and reported cases from normal distributions (Eq. 2). The initial conditions used at the beginning of each simulation are also demonstrated. The mean for the binomial distribution was considered the deterministic value from the previous timestep using the differential equation, and the standard deviation (SD) was a proportion of this value as presented in Table 9. The SIR model is fitted to the data using JAGS and prior distributions.

2 $$ \begin{gathered} S\left[ {t + 1} \right] = S\left[ t \right] - S\left[ t \right] \cdot \left( {1 - e^{{ - \frac{\beta \cdot I}{N}}} } \right)^{1} \left( {1 - e^{{ - \frac{\beta \cdot I}{N}}} } \right)^{S\left[ t \right] - 1} \hfill \\ I\left[ {t + 1} \right]= I\left[ {t} \right]+ S\left[ t \right]*\left( {1 - e^{{{-\!\!-}\frac{\beta \cdot I}{N}}} } \right)^{1} \left( {1 - e^{{{-\!\!-}\frac{\beta \cdot I}{N}}} } \right)^{S\left[ t \right] - 1} - I\left[ t \right]*\left( {1 - e^{ - \gamma } } \right)^{1} \left( {1 - e^{ - \gamma } } \right)^{I\left[ t \right] - 1} \hfill \\ R\left[ {t + 1} \right] \, = R\left[ t \right] + I\left[ t \right]*\left( {1 - e^{ - \gamma } } \right)^{1} \left( {1 - e^{ - \gamma } } \right)^{I\left[ t \right] - 1} \hfill \\ C\left[ t \right]\sim Normal(\mu = \rho \cdot I\left[ t \right]| \sigma = \left( {\rho \cdot I\left[ t \right]} \right)*\tau_{C} ) \hfill \\ \end{gathered} $$

**Table 9:** The parameters of the SIR model that add stochasticity and the distributions these were sampled.

Parameter	Description	Distribution type	Value/interval (CI)	Rationale
τ_C_	Proportion deviation in C	Uniform	(0,1)	Some proportion of the mean to account for white noise
S[0]	Initial susceptible fraction population when year begins	Uniform	(0, 1)	Some amount of the population is susceptible before the outbreak year begins
I [0]	Infected population in last epi week of previous year	N/A	Based on the previous year. Can be continuously predicted	Highest recorded cases in last epi. week is 500
R [0]	Removed population	N/A	N [0]-S [0]-I [0]	N/A

*epi.* Epidemiological, *N/A* not applicable.

The equation shown in (2), for Susceptibles (S), Infected (I) and Removed (R) paramters represents the deterministic solution of its corresponding ODE from (1). When sampling from the binomial distribution, this represents variance. To translate this into a stochastic value, a proportion of this value (τ values) was denoted at the variance. The JAGS model converged on these τ values for susceptibles, infected, and removed in our fit simulations, and the means were used in future simulations. Further descriptions of the parameters, as well as respective prior distributions, are shown in Table 9.

### JAGS model

The reliability of the JAGS model was tested to converge on the parameter values with randomly generated trials of 500-time points each for reflecting the maximum time during application to reported cases (Table 10). The SIR model simulation inputs were all randomly generated from the prior distributions listed above. These were S [0]$$, {\tau }_{C}, \beta ,$$ and $$\rho $$. We set the Population, $$C$$, and reporting rate using data gathered from literature. The parameters that remained constant are displayed as the percentage of trials where the confidence interval successfully converged on the value. The parameters that varied with each time step are represented as mean/SD across 70 trials. In this case, each trial percentage is the proportion of successful convergence across the 500 points. Note that for the purpose of testing, $$\beta $$ was sampled randomly between a (1,5) uniform prior distribution from Liu’s reported minimum and maximum values^33^, as all true values of the transmission parameter would fall approximately within this interval. Each model was run with four parallel chains and 100,000 iterations for adaptation, 100,000 iterations for monitoring, and a thinning interval of 10 for the monitors. This combination showed the lowest SD in the sampled parameter values at each time step, while allowing the computing system to proceed without issue. $$\beta / \gamma $$ is known as the characteristic $${\mathrm{R}}_{0} or basic reproduction number$$ of an infectious disease in the SIR model. There are several estimates as to what this is for dengue fever^8,15^. For our study, the most recent estimates of the time-dependent basic reproduction number, signified as R_0_, from Liu’s spatiotemporal dynamic model in 2018 were used. Then $$\beta $$ can be estimated as $${R}_{0}*y$$ for subsequent testing of the model.

**Table 10:** Results of testing the JAGS model with convergence percentages.

Time-varied parameter	Mean % (SD %) N = 70 trials	Constant parameters	% Convergence
$S$	94.1 (11.1)	τ_C_	94.3
$I$	95.7 (5.0)	S [0]	97.1
$R$	97.3 (6.2)	ρ	100
$\beta$	93.8 (8.4)	N/A	N/A

*SD* standard deviation, *N/A* not applicable.

### Null model

We began testing with the 2019/2020 outbreaks using a constant transmission parameter $\beta$ in our null model. The inputs for $\beta$ are shown in Table 11 and were obtained from Liu’s paper on estimates of the basic reproduction number. Keeping the transmission parameter constant represents no seasonal changes in transmission of the disease, which is biologically the result of increased mosquito populations. Normally distributed JAGS inputs $\beta$ were created based on the tropical environment estimates in Southeast Asian countries, Singapore, and Central/South American countries. The values shown in Table 11 contain data collected in the twenty-first century. The countries in each of these regions are managed by similar government/health agencies and have very similar environments for mosquito breeding Controlling for $\beta$ and all other parameters, the initial susceptible fraction of the population was then estimated annually through JAGS.

**Table 11:** The beta estimates by country.

Country	K: mean (SD)	Distribution
Singapore	2.36 (0.51)	Normal
Honduras	2.11 (0.81)	Normal

All estimates are obtained from literature search.

### Seasonal and climate model

The seasonality of dengue in further application of the model is accounted for in a trigonometric fashion. With the susceptible estimates from the null model, the following trigonometric curve was input into JAGS and estimated the parameters listed in Table 12. This combination of trigonometric curves is traditionally used for modelling the seasonality of infectious diseases.

**Table 12:** Parameters associated with the seasonal trigonometric beta mode with sampling distributions.

Parameter	Description	Distribution	Interval	Rationale
Y	Vertical Shift	Uniform	(0, ${A}_{1 }+{A}_{2}$)	$\beta$ should always remain positive
${A}_{1, }{A}_{2}$	Amplitudes	Uniform	(− 10, 10)	Capture the appropriate $\beta$ intervals
${T}_{1, }{T}_{2}$	Period of function	Uniform	(1, 52)	52 epidemiological weeks in 1 year

3 $$\beta [t]={U+A}_{1}cos(\frac{2\pi t}{{T}_{1}})+{A}_{2}cos(\frac{2\pi t}{{T}_{2}})$$

The time-varying beta values were then used in forward simulations with all other outbreak data from Singapore and Honduras, with the susceptible fractions obtained from the null model. Owing to the time delay between increased transmission and growth of the infected population, lagged regressions were explored between the optimal $\beta$ and the incidence reports. These lagged regressions are useful for the relationship between climate and $\beta$. The final model explores the use of climate variables for estimating K. Weekly values were incorporated in the univariate linear regression, and significant predictors were then incorporated into the multivariate model for assessing interactions between climate variables. The framework for the equation is as follows:

4 $$ \beta \left[ t \right] = C_{1} \cdot climvar_{1} \left[ t \right] + C_{2} \cdot climvar_{2} \left[ t \right] \ldots$$

where ${C}_{n}$ and ${climvar}_{n}$ are the nth coefficient of the nth climate variable, respectively, in the regression. Note that ${climvar}_{n}$ could be any of the following:

$${{climvar}_{n}}^{2}+{climvar}_{n} (quadratic){{,e}^{{climvar}_{n}}(logistic) ,or\,\,simply\,\, {climvar}_{n}}$$

in a general additive model for a known non-linear relationship. For the climate model, the obtained climate reports were first regressed against the cases to evaluate significant predictors at their optimal lag times. The country data were first displayed as one succinct time series, as climate effects need to be continuous, without intervention, considering the susceptible fraction. However, when including initial susceptible fractions, the predictive simulations are presented on a yearly basis. We first correlated El Nino Indicators to monthly incidence in Singapore and Honduras. As mentioned, the El Nino data were manipulated to have the same values for each epidemiological week corresponding to the monthly report. The El Niño variables performed significantly well in regressions, though we choose not to include these results. As mentioned, ENSO indicators were originally monthly reports, and were purposely manipulated to become weekly reports. With weekly climate variables, the same method was used for determining the amount of lag related to the weekly incidence. Lags of 1–40 weeks were used for El Nino indices, and lags of 1–12 weeks were used for weekly fluctuating variables such as precipitation, humidity, etc., which were shown to be appropriate from past research^30^.

### Statistical analyses

To deduce the lag between climate variables, both weekly and monthly, and dengue incidence, the sample cross-correlation function (CCF) was used in R for determining significant lag times. For all climatic variables, four types of regression analysis were employed: linear, quadratic, exponential, and general additive, as studies have shown contrasting regression types between climate variables and incidence. P- values were reported for each of the four types of regression and the best univariate and multivariate fits were displayed. A rejection region of 0.05 was used for climate variables related to incidence. Autocorrelation function tests were implemented to determine lagged correlation between the series of simulated values and the reported case count for both seasonal and climate models. For the SIR model simulations of case count, when compared with reported cases in Singapore and Honduras, auto-correlation values were used for quantifying the relationship, unless specified otherwise. This autocorrelation method shows how closely each prediction model followed the dynamics of the outbreak, and more specifically, how it modeled the “peak” of the cases. All analyses were performed using the R platform and R studio v 3.2.4.

## Conclusion

Early detection strategies for dengue virus outbreaks have considerable impact on the well being of the community. The presented prediction using the seasonal and climate models in this research are very promising. Both models statistically and visually followed the dynamics of the recent and past outbreaks, demonstrating accuracy and reliability and included high variability in the measured parameters. Early detection models that consider pre-outbreak susceptibility measures, in combination with climate variability, provide a reasonable explanation for the 2019–2020 dengue epidemic in Singapore and Honduras, and may serve as a viable predictive tool for future outbreaks in these nations. Future plans should be implemented that focus on improving rapid seroprevalence testing in countries. Regions in non-tropical areas may rely more on the presented seasonal models, as their climate conditions tend to not be optimal for mosquitoes. Small fluctuations in the climate can enhance mosquito breeding. Along with global warming, all countries may soon need to shift their focus towards a climate model-including the United States.

## Data Availability

Code is available upon request from corresponding author. It will be uploaded to the Journal Repository once the paper has been conditionally accepted.
